# The effect of non-pharmaceutical policy interventions on COVID-19 transmission across three cities in Colombia

**DOI:** 10.3389/fpubh.2022.937644

**Published:** 2022-09-15

**Authors:** Adriana Poppe, Dina Maskileyson

**Affiliations:** ^1^Faculty of Management, Economics and Social Sciences, The Institute of Sociology and Social Psychology, University of Cologne, Cologne, Germany; ^2^PMV Research Group, Medical Faculty and University Hospital Cologne, University of Cologne, Cologne, Germany

**Keywords:** COVID-19, non-pharmaceutical policy interventions (NPIs), single-group interrupted time series analysis (ITSA), Colombia, policy evaluation

## Abstract

Governments across the globe have implemented different strategies to handle the COVID-19 pandemic. A national mandatory quarantine was the most applied policy tool. While there are studies that tested the effectiveness of a national mandatory quarantine, the question about the effectiveness of additional quarantine policies is not yet answered. In this study we focus on three large cities in Colombia (Bogota, Medellin and Cali) with similar socio-economic conditions but made use of different COVID-19 prevention measures. We examine whether different non-pharmaceutical policy interventions (NPIs) conducted in these three cities are effective against the spread of the COVID-19 pandemic. We inspect the effect of the quarantine policies restricting exit from home by sex, ID number, whereby only Bogota implemented the restriction to leave the home according to sex followed by a restriction according to ID number, and Medellin and Cali implemented a restriction by ID number only. Data for the analysis are obtained from the National Administrative Department of Statistics of Colombia [Departamento Administrativo Nacional de Estadística (DANE)]. The data on pandemic severity is measured by the number of confirmed COVID-19 cases per city. We conduct single-group interrupted time series analysis (ITSA) to examine differences in the extent of the pandemic severity in Bogota, Medellin and Cali. We found that NPIs in all three Colombian cities had a positive effect on slowing the spread of the pandemic.

## Introduction

A new coronavirus (COVID-19) emerged on December 12, 2019, in Wuhan, China (1). The first confirmed case of COVID-19 was diagnosed in Colombia on March 6, 2020, in the capital city of Bogota. In the following weeks, the virus has spread very quickly around the country. In response to this and in addition to measures implemented by the national government, regional governments have introduced different non-pharmaceutical policy interventions (NPIs) to lower the infection incidence curve (2). Specifically, these NPIs included *pico y cédula* (ID) and *pico y género* (sex) and defined days on which people are allowed to do errands and buy necessities. *Pico y cédula* (ID) NPI divided days according to even and odd dates and even and odd final digit of identification documents (IDs). *Pico y género* (sex) NPI allowed people to leave their home based on their sex (e.g., women were allowed to go out on even and men on odd date).

To date, empirical evidence of the effectiveness of different NPI types in terms of the morbidity and mortality due to COVID-19 remains inconsistent (3–8). Furthermore, to the best of our knowledge, no one has yet investigated whether the effect of the implemented NPIs on COVID-19 spread differs among different cities in the same county in the Latin American context. Therefore, this study aims to close this research gap by analyzing the influence of city-specific NPIs on the distribution of COVID-19 cases. We specifically evaluate the success of the implemented policies in the Colombian cities of Bogota, Cali, and Medellin.

The analysis enables us to provide a 2 fold contribution to the literature. First, we expand the knowledge on the policy effectiveness during the COVID-19 pandemic in the context of Colombia in particular as well as in the Latin American and the global context in general. To the best of our knowledge, this is the first study that examines the effectiveness of pandemic prevention policies such as *pico y cédula* (ID) and *pico y género* (sex). The knowledge obtained from this study might be helpful for understanding and planning future prevention and control measures to combat epidemics and pandemics not only in Colombia. The results can and should be applied to populations with socioeconomic characteristics similar to the population of Colombia.

Policy makers tend to rely on the knowledge gained during the course of previous epidemics and pandemics to implement effective measures designed to stop the spread of the virus (9, 10). In the case of a new virus, references to similar viruses, such as severe acute respiratory syndrome (SARS), are made in order to evaluate possible NPIs aiming to flatten the curve (9). During the SARS outbreak, social distancing and hand hygiene were implemented as measures for reducing the virus spread with the objective to narrow the gap between medical need and available supply of treatments (9, 11). In addition, measures such as isolation, quarantine and social distancing were implemented in the most affected countries to control the person-to-person transmission of SARS (9).

There is an emerging body of studies that examine the efficiency of different measures to stop the spread of COVID-19 [for details see systematic review by Perra (12)]. For example, Liu et al. (8) have investigated impact of NPIs on COVID-19 transmission across 130 countries and territories. They used longitudinal regression to estimate the effectiveness of 13 categories of NPIs in reducing COVID-19 transmission using data from January to June 2020. The authors concluded that understanding the impact that specific NPIs have had on COVID-19 transmission is complicated by temporal clustering, time-dependent variation in effects, and differences in NPI intensity. However, the effectiveness of school closure and internal movement restrictions appeared robust across different model specifications, with some evidence that other NPIs may also be effective under particular conditions. Therefore, Liu et al. (8) argue that many, although not all, actions policymakers are taking to respond to the COVID-19 pandemic are effective. Another study by Yang et al. (13) tested the effectiveness of two major NPIs—lockdown-like measures that reduce contact rates and universal masking in New York City. Using data from the 2020 spring pandemic wave, they found that face covering can substantially reduce transmission when lockdown-like measures are lifted but by itself may be insufficient to control COVID-19 transmission (13).

Díaz-Castro et al. (14) recently examined the impact of policies that have been implemented in response to the COVID-19 pandemic on the velocity of viral transmission, as reflected by the doubling time, considering the mobility and sociodemographic characteristics across the 32 Mexican states. Their results revealed that health policies had an effect on slowing the pandemic's propagation, but population density and mobility played a fundamental role (14). Another study by de Figueiredo et al. (3) focused on the Hubei and Guangdong provinces in China using the number of COVID-19 cases per 10,000 inhabitants to estimate the pandemic spread between January 23, 2020, and March 12, 2020 (3). The longitudinal effects of an intervention on a were outcome are analyzed using the Interrupted time series analysis (ITSA) (3). The absence of the intervention (contractual) and the trend found after the intervention are considered (3). The authors have shown that the social distancing measures in the two provinces were effective in reducing incidences and mortality rates of COVID-19 (3). Castex et al. (15) examined the effectiveness of lockdown policies in 132 countries using data provided by the Oxford COVID-19 Government Response Tracker. They demonstrated that the effectiveness of lockdown policies declines with GDP per capita, population density and surface area of the country and increases with health expenditure and lower physician-to-population ratio (15). In the context of Latin America, the impact of the implementation of a general mandatory quarantine and the implementation of mask obligation in public spaces has been tested in Colombia, Costa Rica, Peru, Ecuador, Mexico and Chile using ITSA (7). A curve-flattening effect of the general mandatory quarantine was found in Colombia but not in Ecuador or Peru. Using a similar methodological approach, Silva et al. tested the effectiveness of implemented social distancing policies in four Brazilian cities. The results indicated a statistically significant decrease in new confirmed cases in all cities tested after the implementation of a lockdown (16). González-Bustamante (17) has examined non-pharmaceutical interventions related with measures of social distancing, closure of schools, workplaces, public transport and restrictions on meetings and national and international travel in eight South American countries: Argentina, Bolivia, Brazil, Chile, Colombia, Paraguay, Peru, and Uruguay. His results revealed that only Uruguay and Paraguay have managed to control the pandemic by mid-May, while Brazil and Peru have faced very adverse scenarios. The author has emphasized that the effectiveness of the NPIs needs to be studied in greater depth, considering diverse institutional and sociocultural factors. This article therefore aims to expand the knowledge on the effectiveness of the NPIs that were not yet studied: *pico y cédula* (ID) and *pico y género* (sex).

The policy *pico y cédula* (ID) was implemented in Medellin on April 2, in Cali on April 6 and in Bogota on June 16. Pico y cédula determines, according to an individual's ID number, on which day a person is allowed to leave the house for errands. Persons who have an odd number as their final ID digit are only allowed to leave the house on odd-numbered days, persons with even numbers are only allowed to leave on even-numbered days. This rule was not applicable for people leaving their homes for working purposes. Notably, of the three large cities investigated here, Bogota is the only one that introduced the policy *pico y género* (sex). This policy determines who is allowed to leave the house for shopping or similar activities according to sex (e.g., women were allowed to go out on even and men on odd dates^1^). This policy was introduced on April 10 and expired on May 11, 2020. Both policies have been implemented as an extension to the general mandatory quarantine implemented by the government.

## Materials and methods

### Data

The data on the confirmed COVID-19 cases used in this study are obtained from the National Administrative Department of Statistics of Colombia—DANE. Specifically, COVID-19 data is reported directly to the National Institute of Health of Colombia, which reports the number of new positive cases to DANE, by the laboratory which processes the polymerase chain reaction (PCR) tests on a daily basis. The data is provided by the Colombian Ministry of Health and is separated by the 32 departments that make up the country (19). Based on the population size as well as implemented policies three cities have been selected for analysis. Hence, sample for this study includes the three Colombian cities with the highest population and similar socio-economic conditions (20). The city of Bogota is part of the department Bogota D.C., Cali is the capital of Valle de Cauca and Medellin is the capital of Antioquia. No data on the number of PCR tests that were administered on the city-level could be found (see Table 1 for the number of administered PCR tests in the three departments included in this study).

**Table 1:** Number of COVID-19 PCR tests per 100,000 inhabitants, by department.

	**Population total (2018)**	**May 10th**	**June 10th**	**July 10th**	**August 10th**	**September 10th**	**October 10th**	**November 10th**	**December 10th**
Bogota D.C.	7,149,540	647.26	1811.53	4017.84	8545.92	13313.57	16574.33	20503.65	24280.92
Valle de Cauca	3,762,229	437.16	1038.51	2908.33	3953.69	5660.58	6838.90	8464.88	10183.70
Antioquia	5,931,492	356.76	923.80	1336.63	4350.19	6137.76	7684.36	9929.07	11657.71
Total (Colombia)	43,835,324	345.37	1015.01	2209.33	4431.88	6543.33	8240.58	10273.77	12279.89

These are, following the 2018 census, Bogota as the capital district with 7,149,540 inhabitants, Medellin with 2,359,801 inhabitants and Cali with 1,811,385 inhabitants (20).

### Variables

The dependent variable is the number of confirmed COVID-19 cases per day per 1,000 inhabitants in each city. The number of new confirmed COVID-19 cases is based on the reported day of diagnose by positive PCR tests by laboratories to DANE. Missing data have been found in 408 cases. In 407 cases, the first day when symptoms appeared has been used as proxy for the day of the diagnosis. In one case, the day of the beginning of the symptoms was not available, and therefore, the day the case was reported to the web/online report was used. The analysis of the cases without missing data showed that there were only a negligible date differences between the diagnosis date and the date reported to the web/online report. In order to control for the exponential growth in the daily confirmed cases, we transformed this variable as a natural logarithm. The time elapsed since the start of the pandemic is measured in days. We analyzed the time period of 155 days after the first confirmed case in each city. Hence, the starting time point of the analyses varies among the cities, but the length of time is set equal among the cities. The date of the first included case for Bogota was March 6, for Cali—March 13, and for Medellin—March 9. Due to expected delay in the effect of the implemented policy on the distribution of confirmed COVID-19 cases, the interruption time-point of the analyses is 14 days after the actual implementation of the policy in order to control for an expected delay between implementation of the policy and the effect on the number of the new cases (7).

### Methods

We conducted ITSA to examine whether the implementation of a certain policy has exerted a decreasing effect on the distribution of the cases in each city. ITSA has often been used to estimate, for instance, policy impacts (4, 21) or the effects of health care interventions (22). This method allowed us to evaluate the policy impact before and after the implementation without having a control group. That is, a single-group ITSA is designed without a comparable control group, it rather projects the pre-intervention trend into the treatment period, which serves as the counterfactual (23). The model is based on the following equation (23, 24):


(1) $$\begin{array}{r} Y_{t}=\beta_{0}+\beta_{1}T_{t}+\beta_{2}X_{t}+\beta_{3}X_{t}T_{t}+o \end{array}$$

The outcome variable measured at each time point is represented by *Y*_*t*_. The starting level (intercept) of the outcome variable is represented by β_0_. β_1_ is the slope of the trend of new cases before the start of the intervention, the immediately occurring change in the level of the outcome after the start of the intervention is represented by β_2_. β_3_ is the difference between pre-intervention and post-intervention slopes of the trend of confirmed cases per 1,000 inhabitants. Using single-group ITSA, the pre-intervention trend is projected into the treatment period which serves as counterfactual (23). Based on an expected incubation time (25) this study focuses mainly on the difference between pre-intervention and post-intervention slopes (β_3_) rather than the immediate occurring change in the level of the new cases per day (β_2_).

All analyses are conducted using STATA 15.1. The ITSA was conducted using the STATA command *itsa* (23). To account for autocorrelation and heteroscedasticity in the error terms, Newey-West estimators were used (26). The lags of the serial correlation in the data were specified with the STATA command *actest* (27). The command performs a Cumby-Huizinga general test for autocorrelation in time series data, with the null hypothesis that serial correlation exists in the time series, but it dies out at a known finite lag (*q* > 0) (27). The lag in which the series correlation dies out was included into the ITSA model to control for it.

## Results

### Descriptive overview

The cumulative number of PCR tests per 100,000 inhabitants per department under investigation are displayed in Table 1. The table show that the difference between the proportion of tests performed in the departments and the proportion of the population living in them varies. Thus, when considering the results of the analysis, the different percentages of tests performed must be taken into account.

**Figure 1:**
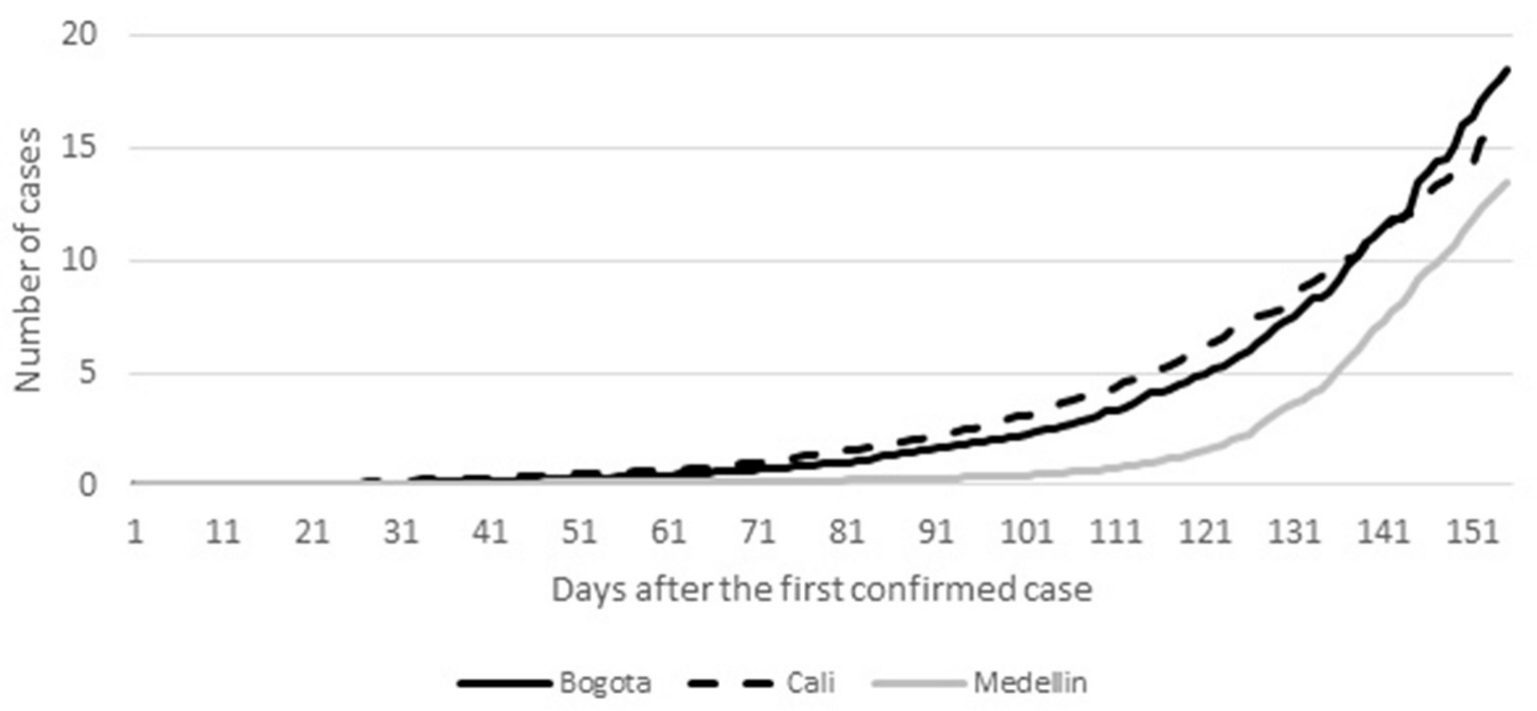
Distribution of the COVID-19 confirmed cases per 1,000 inhabitants by city.

Figure 1 demonstrates the distribution of the confirmed COVID-19 cases per 1,000 inhabitants by city. It includes the day of the first confirmed case per city and the ensuing 155 days. By the end of the observation, Bogota had the highest number of confirmed cases per 1,000 inhabitants. Cali was the city with the second highest number of confirmed cases per 1,000 inhabitants by the end of the observation period.

### Testing the effect of *pico y género* (sex) and *pico y cédula* (ID) across the three cities

We conducted single ITSA to examine whether and to what extent the city-specific NPIs *pico y género* (sex) and *pico y cédula* (ID) have a decreasing effect on the distribution of confirmed COVID-19 cases.

The results of the analyses are displayed in Table 2. As Bogota was the only city to implement both the *pico y género* (sex) and *pico y cédula* (ID) NPIs two models for Bogota [Model 1a for *pico y género* (sex) and Model 1b for *pico y cédula* (ID)] and one model for each Cali (Model 2) and Medellin (Model 3) have been estimated.

**Table 2:** Single ITSA predicting the effect of peak and sex/peak and id on the COVID-19 infection rate, by city; the interruption time-point of the analysis is 14 days after the actual implementation of the policy.

	**Bogota (peak and sex)**		**Bogota (peak and ID)**		**Cali (peak and ID)**		**Medellin (peak and ID)**	
	**Model 1a**		**Model 1b**		**Model 2**		**Model 3**	
	β	**SE**	β	**SE**	β	**SE**	β	**SE**
Pre-intervention								
Intercept	−7.311^***^	0.550	−5.402^***^	0.879	−6.315^***^	0.369	−6.868^***^	0.251
Slope	0.149^***^	0.019	0.068^***^	0.011	0.159^***^	0.017	0.134^***^	0.012
Post-intervention								
Intercept	−1.164^*^	0.512	−0.992	0.559	−0.837^*^	0.324	−1.328^**^	0.474
Difference between pre- and post-intervention slopes	−0.109^***^	0.019	−0.028^*^	0.012	−0.124^***^	0.175	−0.088^***^	0.011

Significance level: *p < 0.05, **p < 0.01, ***p < 0.001; SE, standard error. All models are estimated 14 days after the implementation of the intervention as the interruption time-point of the analyses to control for a lag between infection, symptoms and PCR test results.

**Figure 2:**
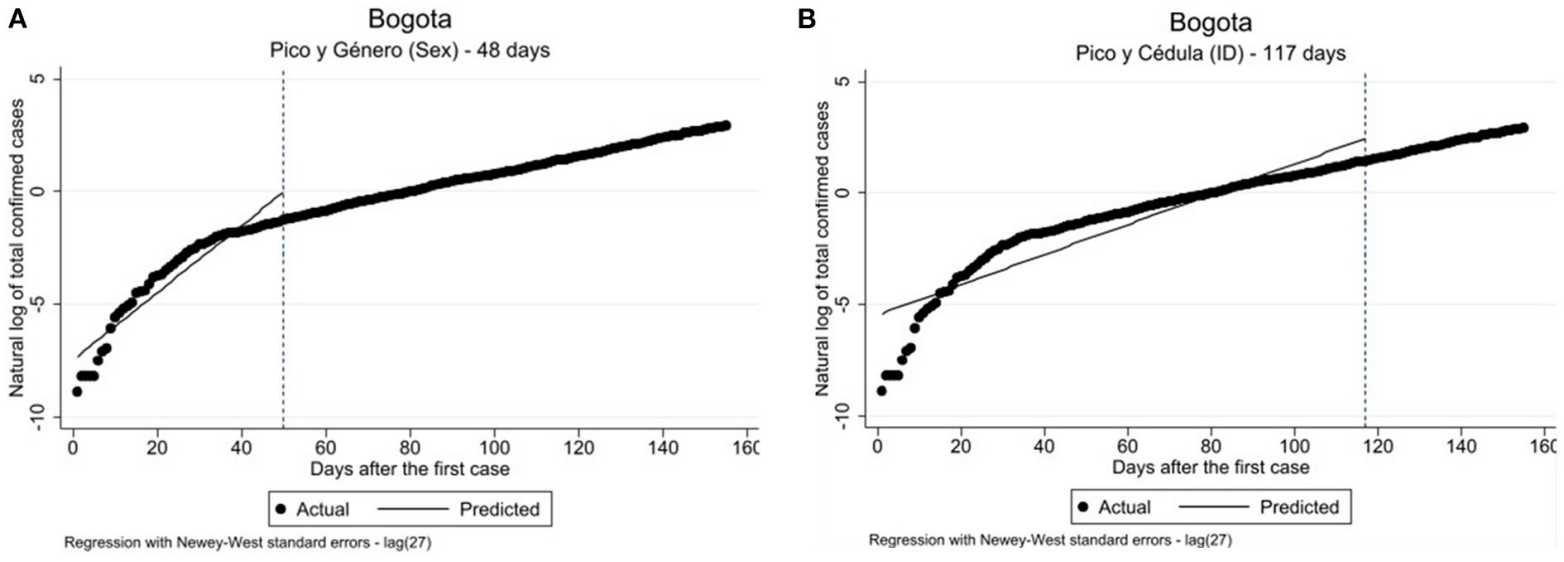
**(A)** Single ITSA predicting the effect of peak and sex/peak and id on the COVID-19 infection rate, by city; the interruption time-point of the analysis is 14 days after the actual implementation of the policy. **(B)** Single ITSA predicting the effect of peak and ID on the COVID-19 infection rate in Bogota; the interruption time-point of the analysis is 14 days after the actual implementation of the policy.

Model 1a tested the effect of the policy *pico y género* (sex) on the number of confirmed cases reported by the PCR -test laboratory to DANE 14 days after its implementation [*F*_(3, 151)_: 11676.9, *p* ≤ 0.000]. The 14 days delay was selected to control for an estimated delay of the policy based on the COVID-19 incubation period. Both the pre-intervention intercept [ß = −7.311, exp(ß) = 0.550, *p* = ≤ 0.001] and pre-intervention slope [ß = 0.163, exp(ß) = 1.17, *p* = ≤ 0.001] indicate a statistically significant increase in the prevalence of COVID-19 cases before the implementation of the policy. The post-intervention intercept is negative and statistically significant [ß = −1.164, exp(ß) = 0.512, *p* = ≤ 0.05]. Moreover, difference between pre- and post-intervention slopes is negative and statistically significant [ß = −0.109, exp(ß) = 0.018, *p* = ≤0.001], indicating a decrease in the COVID-19 prevalence rate over time. Thus, a curve-flattening effect of the policy *pico y género* (sex) implemented in Bogota is verified by the analysis 14 days after the implementation and over time. The visual verification of these results is presented in Figure 2A.

Model 1b presented in Table 2 shows the results of the examination of the *pico y cédula* (ID) policy effect with the interruption time-point set at 120 days (i.e., 14 days after the policy implementation) [*F*_(3, 151)_: 2333.44; *p* ≤ 0.000]. Figure 2B displays the visualization of the model. The results reveal a similar trend in the *pico y cédula* (ID) policy effect as compared to the *pico y género* (sex) policy effect described above. Therefore, it will not be discussed in detail. Both policy interventions in Bogota had curve-flattening effects on the distribution of COVID-19 case numbers as demonstrated by the ITSA coefficients for Models 1a and 1b (see Table 2).

**Figure 3:**
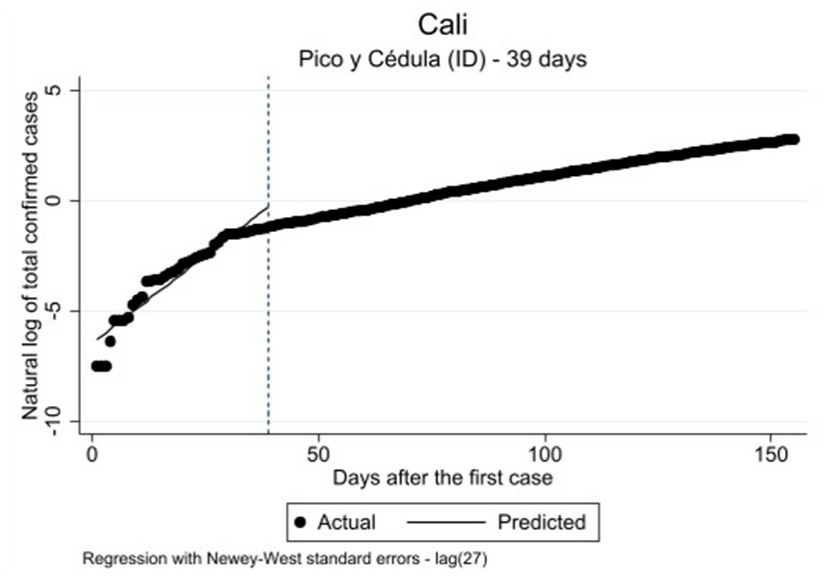
Single ITSA predicting the effect of peak and ID on the COVID-19 infection rate in Cali; the interruption time-point of the analysis is 14 days after the actual implementation of the policy.

The results for Model 2, the *pico y cédula* (ID) policy effect implemented in Cali, are provided in Table 2 [*F*_(3, 151)_: 865.34; *p* ≤ 0.000]. We found a decreasing statistically significant change in the post-intervention intercept [ß = −0.837, exp(ß) = 0.324, *p* ≥ 0.05]. Similar to the results for Bogota, we also fund a statistically significant difference between pre- and post-intervention slopes [ß = −0.124, exp(ß) = 0.175, *p* ≤ 0.001], signifying a statistically significant decrease in COVID-19 cases throughout the duration of the observation period. Therefore, the results demonstrate a curve-flattening effect of the *pico y cédula* (ID) NPI. Figure 3 illustrates the lower number of COVID-19 cases in Cali at the end of the observation period than was predicted by the analysis.

**Figure 4:**
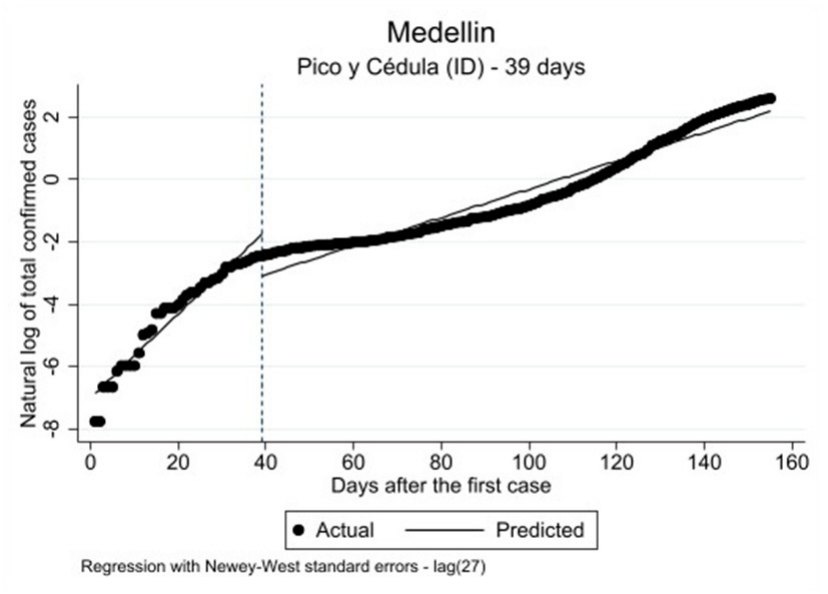
Single ITSA predicting the effect of peak and ID on the COVID-19 infection rate in Medellin; the interruption time-point of the analysis is 14 days after the actual implementation of the policy.

As the results provided in both the regression Model 3 (Table 2) and Figure 4 indicate, the effect of the *pico y cédula* (ID) policy in Medellin is similar to the effect of the *pico y género* (sex) policy introduced in Bogota (Model 1a, Table 2) [*F*_(3, 151)_: 226.06, *p* ≤ 0.000]. Therefore, it will not be discussed in detail.

## Discussion and conclusions

Latin America has been severely affected by the COVID-19 outbreak. The government response to COVID-19 in this region has been diverse in nature and mixed in terms of the effectiveness [(e.g., (28, 29)]. Colombia is a typical third-most-populous country in Latin American whose population is 50 million inhabitants. Colombia's response to the COVID-19 emergency displays an interesting case study of unitary but decentralized administrative approach (30). Namely, Colombian government has been taking measures simultaneously on the national, state, and local levels to prevent transmission of the virus. In this study, we have focused on the political response to the COVID-19 pandemic on the local level in Colombia and focused on the period between the occurrence of the first case in March 2020 until August 2020 (155 days after the first case in each city).

Many policy analyses of the COVID-19 outbreak have focused on the measures implemented to contain the spread of the disease and on how effective these measures are in reducing the number of new infections and deaths. These studies, however, are largely limited to high income countries (e.g., (31)) with a few exceptions in middle income countries [e.g., Castex et al. (15) and Silva et al. (16)] that are restricted by the lack of data. Real-time analysis of epidemiological data as well as estimations of the preventative measures in Latin America are very limited and lacking the level of detail required to increase situational awareness and to guide policy decisions in this region. Previous studies on middle income countries have mainly examined the effect of the most common confinement policies (e.g., general mandatory quarantine, social distancing) [see for example, González-Bustamante (17)—for Argentina see; Poppe (7), Castex et al. (15)—for Colombia, Costa Rica, Peru, Ecuador, Mexico, Chile, and Silva et al. (16)—for Brazil]. This study investigated the impacts of two different NPIs adopted by the Colombian government on the city level to contain the COVID-19 spread. Specifically, we evaluated the policies implemented in the three largest cities in Colombia: Bogota, Cali and Medellin. Notably, all three cities had similar pre-conditions regarding the restrictions to leave the house because in addition to NPIs the government of Colombia has implemented general mandatory quarantine across the country. Examining locally implemented policies in the three largest cities of this country allowed us to test administrative efforts on the local level (i.e., city level) and not only on the national and state levels. By doing so, we conducted single ITSA using the data on confirmed COVID-19 case numbers per 1,000 inhabitants in each city available from DANE. We tested the city-specific quarantine policies regulating exit from home by sex and ID number. Specifically, we estimated the models for the *pico y género* (sex) and the *pico y cédula* (ID) restrictive policies for the city of Bogota, and the *pico y cédula* (ID) policy for both Medellin and Cali.

Therefore, current study expands the knowledge on the effectiveness of the NPIs that were not yet studied: pico y cédula (ID) and pico y género (sex). The results of the previous studies on the national level policies outcomes are somewhat inconsistent. Some of these studies revealed that the common confinement measures were effective in reducing the spread of the COVID-19 pandemic. For example, Poppe (7) showed a curve-flattening effect of the general mandatory quarantine in Colombia. Silva et al. (16) revealed that a lockdown led to a statistically significant decrease in new confirmed cases in four Brazilian cities. Other studies found only partial support for this evidence. In his study on South American countries, González-Bustamante (17) found, for example, that general confinement measures were effective only in two (Uruguay and Paraguay) out of eight countries. In line with previous studies demonstrating positive outcomes of the general confinement measures, our results reveal that pico y género (sex) and the pico y cédula (ID) were effective in three Colombian cities. Specifically, the results for Bogota revealed that both versions of the policy have a curve-flattening effect over time. We found statistically significant differences between the pre- and post-intervention intercept, as well as statistically significant decrease in the difference between pre-and post-intervention slope. In other words, in the long term, both NPI policies implemented in Bogota helped to decrease the COVID-19 case numbers compared to what would have happened had they not been implemented. The analytical results for Cali and Medellin were similar to those of Bogota. We did not find any differences in the infection rates by sex.

The findings presented in this study make two distinct contributions to the COVID-19 policy literature. On the empirical side, the present research provides insights into the effectiveness of the local level policies—pico y género (sex) and the pico y cédula (ID). From the theoretical perspective, this study contributes to understanding of the importance of the combination of national policies and local decisions to mitigate the impacts of the COVID-19 pandemic. Colombian response to the COVID-19 pandemic demonstrates that the dual system might work to build a coordinated and effective intergovernmental strategy. The knowledge resulting from this study may be beneficial for formulating new COVID-19 policy in other countries of Latin America. The results can be applied to populations with socioeconomic characteristics similar to the study population. Policymakers in both low- and middle-income countries with limited budged as well high-income countries with low COVID-19 vaccination level should consider a complex multilevel governance structure. Local level policies can be beneficial as the basis of public health interventions due to their high cost-effectiveness and high speed of implementation, and can help to be better prepared for potential future pandemics.

Several limitations must be borne in mind when interpreting the findings of this study. Firstly, only reported and confirmed cases could be included in the analysis. Thus, this paper only refers to reported cases of COVID-19 published for the respective cities. In this sense, the number of unreported cases, which is estimated differently depending on the reproductive value, cannot be included (32). The possibility of a bias due to a high number of unreported cases exists, depending on the testing frequency of the cities. As data on number of performed PCR tests by city are currently lacking, it was not possible to control for this possible bias [cf. (33)]. It must also be noted that some segments of the population might be underrepresented in the data. For example, people of lower socio-economic status might have difficulties with access to testing (6), which may be explained by general difficulties in access to healthcare. In an attempt to overcome this limitation, in this study we focused only on the cities with similar socio-economic conditions and similar population size and density. In addition, the impact of other policies can only be monitored to a limited extent in the present analysis.

Notwithstanding these limitations, the current study was one of the first to assess the effectiveness of underexamined local policy measures implemented during the COVID-19 pandemic, focusing on outbreak in a developing country in a systematic way and using state of the art empirical methods. Our results confirm the effectiveness of the implemented NPI policies. All of them without exception were successful in reducing the COVID-19 growth rate. Understanding the effectiveness of anti-COVID-19 specific policies provides policy-makers with the necessary knowledge to enable them to better understand the policies and act accordingly.

Although this research succeeded in reaching its aims, future investigations of the COVID-19 policy would benefit from evaluating their impact on the social inequalities. It is necessary to consider the multiple consequences of this NPIs, especially in low- and middle-income countries with higher poverty and unemployment rates. The knowledge obtained from these studies may help to prepare the population for the future COVID-19 waves and for the future potential pandemics.

## Data availability statement

Publicly available datasets were analyzed in this study. The dataset was provided by DANE. The dataset can be generated from the following websites: https://coronavirus.softsimulation.com/?rb=m&amp;c$=$11001; https://www.datos.gov.co/Salud-y-Protecci-n-Social/Casos-positivos-de-COVID-19-en-Colombia/gt2j-8ykr/data.

## Author contributions

AP and DM contributed to conception and design of the study, wrote the first draft of the manuscript, and wrote sections of the manuscript. AP organized the database and performed the statistical analysis. Both authors contributed to manuscript revision, read, and approved the submitted version.

## Funding

We acknowledge support for the Article Processing Charge from the DFG (German Research Foundation, 491454339).

## Conflict of interest

The authors declare that the research was conducted in the absence of any commercial or financial relationships that could be construed as a potential conflict of interest.

## Publisher's note

All claims expressed in this article are solely those of the authors and do not necessarily represent those of their affiliated organizations, or those of the publisher, the editors and the reviewers. Any product that may be evaluated in this article, or claim that may be made by its manufacturer, is not guaranteed or endorsed by the publisher.

## Footnotes

^1^Non-binary persons and people who do not identify with their sex assigned at birth were supposed to choose one day to go out (18). Transgender individuals were expected to choose the day assigned to their identified sex (18).

## Supplementary material

Supplementary Figure 1 Distribution of COVID-19 confirmed cases by sex in Bogota.

Supplementary Figure 2 Distribution of the COVID-19 confirmed cases by sex in Cali.

Supplementary Figure 3 Distribution of the COVID-19 confirmed cases by sex in Medellin.
