# Supplementary figures and images for: The effect of non-pharmaceutical policy interventions on COVID-19 transmission across three cities in Colombia
Source: Front Public Health. 2022 Sep 15;10:937644. doi: 10.3389/fpubh.2022.937644 (PMC9521598; doi:10.3389/fpubh.2022.937644)

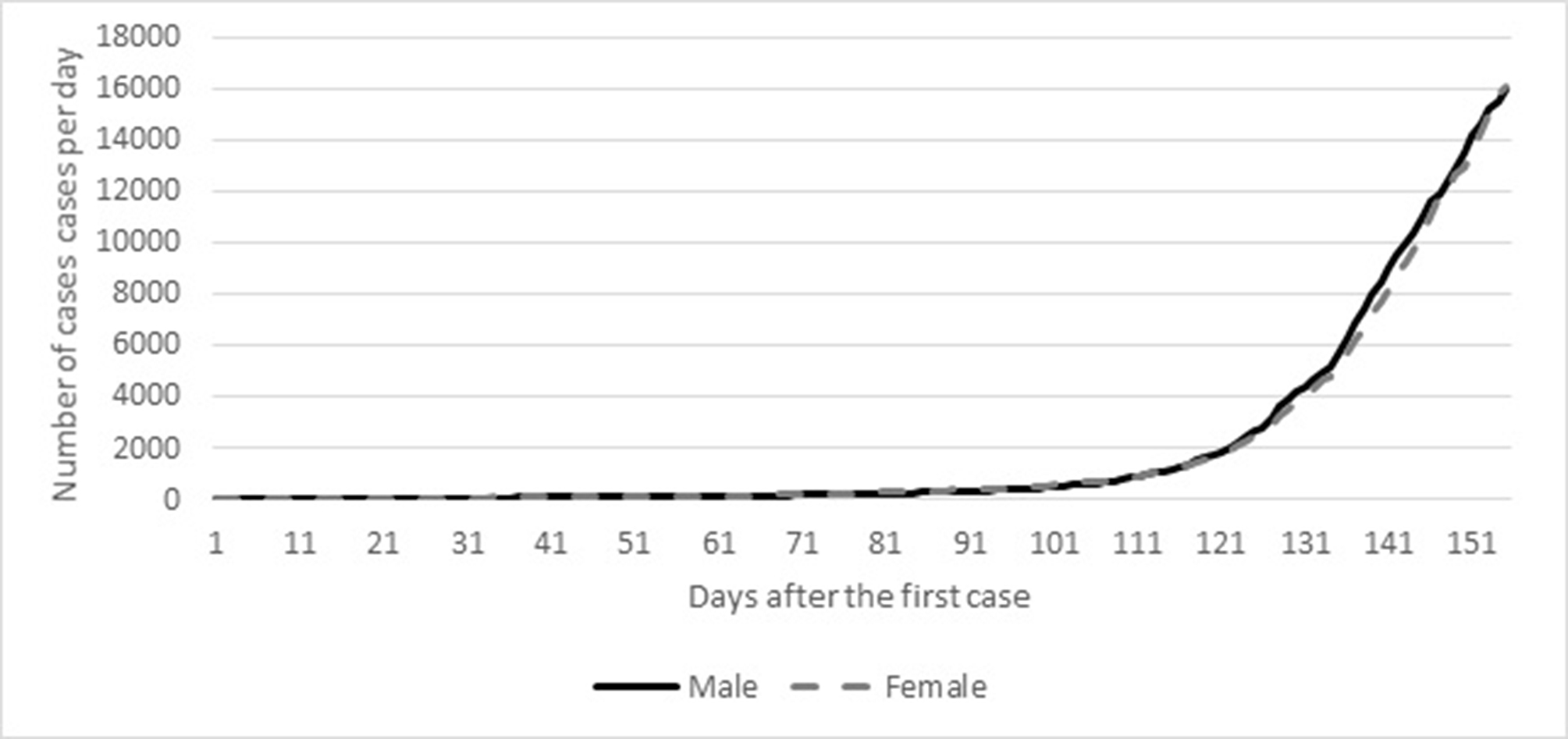

Supplement: Supplementary Figure 1 — Distribution of COVID-19 confirmed cases by sex in Bogota. [file Image_1.JPEG]

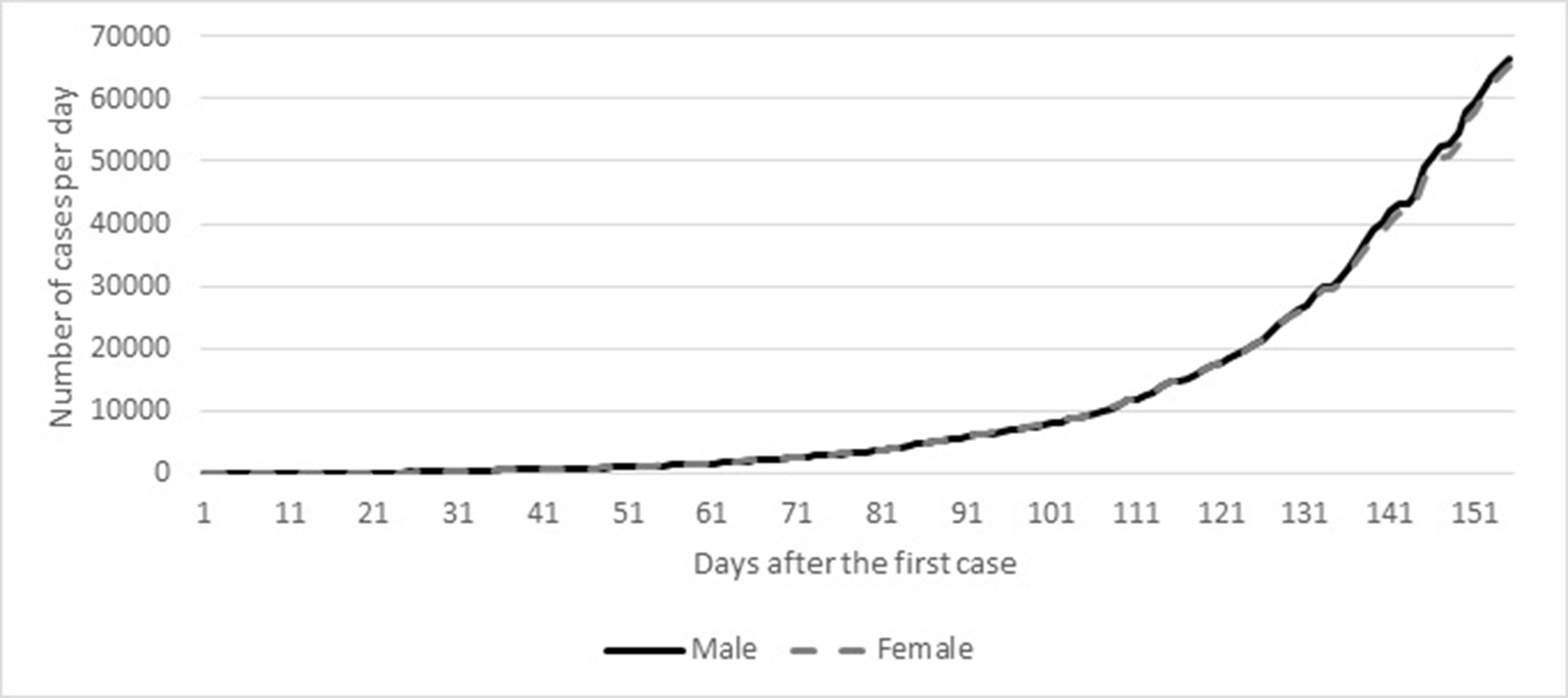

Supplement: Supplementary Figure 2 — Distribution of the COVID-19 confirmed cases by sex in Cali. [file Image_2.JPEG]

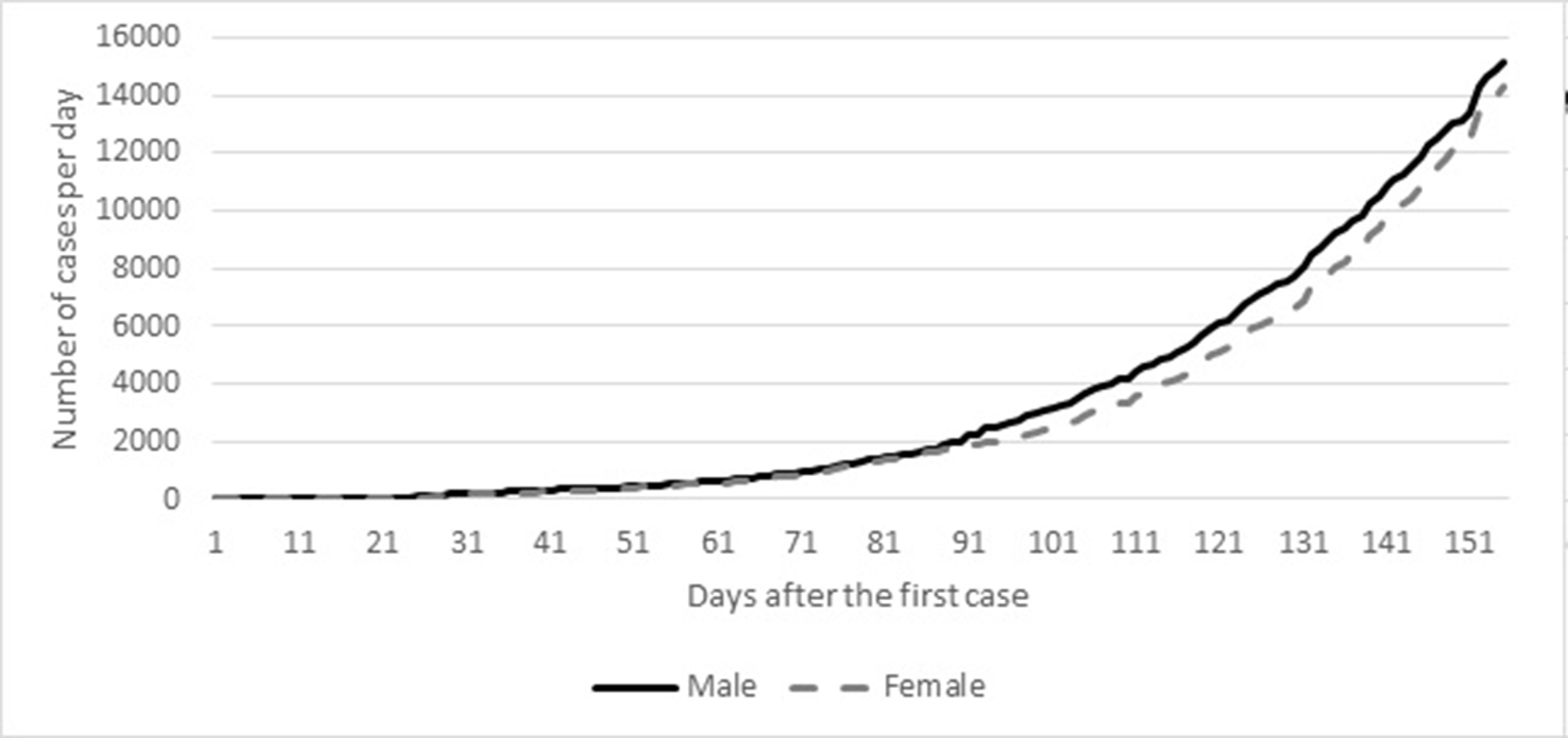

Supplement: Supplementary Figure 3 — Distribution of the COVID-19 confirmed cases by sex in Medellin. [file Image_3.JPEG]
